# Comparison of the biological properties between 3D-printed and decellularized tracheal grafts

**DOI:** 10.1007/s00449-023-02867-4

**Published:** 2023-05-12

**Authors:** Yao Wang, Jianfeng Li, Jun Qian, Yunhao Sun, Jianning Xu, Jian Sun

**Affiliations:** 1 Department of Cardiothoracic Surgery, Yancheng First Hospital, Affiliated Hospital of Nanjing University Medical School, Yancheng, 224005 China; 2 Yizheng Hospital, Drum Tower Hospital Group of Nanjing, Yizheng, 211900 China

**Keywords:** Tissue-engineered trachea, Biomechanical property, Biocompatibility, Decellularized tracheal graft, 3D-Printed tracheal graft

## Abstract

This study sought to characterize the differences between the 3D-printed and decellularized tracheal grafts, providing the basis for the synthesis of the more reasonable and effective tissue-engineered trachea. We compared the biomechanical properties and biocompatibility of the 3D-printed tracheal graft and decellularized tracheal graft in vitro and evaluated the biocompatibility, immune rejection and inflammation of the two materials through in vivo implantation experiments. Compared with the decellularized tracheal graft, the 3D-printed tracheal graft was associated with obviously higher biomechanical properties. The results demonstrated enhanced growth of BMSCs in the decellularized tracheal graft compared to the 3D-printed one when co-culture with two tracheal graft groups. Moreover, the CCK-8 assay demonstrated significant cell proliferation on the decellularized tracheal graft. Serum IgG and IgM measured in vivo by implantation testing indicated that the 3D-Printed tracheal graft exhibited the most significant inflammatory response. HE staining indicated that the inflammatory response in the 3D-printed tracheal graft consisted mainly of eosinophils, while little inflammatory cell infiltrates were observed in the decellularized tracheal graft. CD68 immunohistochemical analysis indicated that the infiltration of macrophages was not significant in both tracheal grafts. Our findings suggest that the biomechanical properties of the 3D-printed tracheal grafts are better than the decellularized tracheal grafts. Nonetheless, the decellularized tracheal graft exhibited better biocompatibility than the 3D-printed tracheal graft.

## Introduction

End-to-end anastomosis after resection of the diseased tracheal segment is generally considered the current gold standard for tracheal reconstruction [1–4]. However, it is only indicated when the diseased trachea does not exceed one-half the length of the whole trachea in adults or one-third in children [5]. When the diseased trachea exceeds the maximum length, tracheal reconstruction is difficult since it is not a simple cylindrical ventilation tube but a sophisticated multi-layer structure [6]. The trachea contains 15–20 C-shaped cartilage rings. The inner surface of the trachea is covered with ciliated epithelium, and the outer surface contains connective tissues such as smooth muscle and blood vessels. The tracheal cartilage importantly maintains the cylindrical shape of the trachea to prevent the trachea from collapsing, while the cilia in the respiratory epithelium on the inner surface of the trachea play an important role in cleaning the trachea. The connective tissue around the tracheal cartilage ensures the mechanical movement of the trachea, involving contraction and expansion [7, 8]. Accordingly, it is extremely challenging to completely reconstruct such a complex multi-layer structure and simulate its function. However, the tissue-engineered trachea has brought increased hope for tracheal reconstruction [9]. It effectively links seed cells, substrate and cytokines to repair related tissue and reconstruct organs [10]. As the main framework of the tissue-engineered trachea and the soil for seed cell growth, the scaffold material is the basis for successfully synthesizing tissue-engineered tracheas [11, 12]. Primary allogeneic tracheal matrix materials and synthetic materials have been widely used for tissue-engineered tracheal scaffolds [13, 14].

As a bioabsorbable polymer, polycaprolactone (PCL) has a low melting point (60 ℃) and good thermoplasticity; accordingly, it is easy to process and mold. Importantly, PCL can be naturally degraded in vivo and metabolized in vitro at a suitable rate[15]. In addition, due to its good biomechanical properties, PCL has been approved by FDA in the United States for biomedicine and tissue engineering [16]. Through experiments, our research group screened out that the 3D-Printed PCL scaffold with a pore size of 200 µm is most beneficial to cell adhesion and proliferation [17]. Hydrolysis, amination and nano-material modification were used to improve the cytotropism of the 3D-printed tracheal scaffold and enhance its adhesion to cells. This study aimed to compare the mechanical properties and biocompatibility of two kinds of tissue-engineered tracheal matrix materials to provide a basis for constructing a more effective and reasonable tissue-engineered tracheal.

## Materials and methods

All the experiments were carried out based on the Guide for the Care and Use of animals, issued by the People’s Republic of China government in 2006. The study protocol was approved by the Ethics Committee of Yancheng First People’s Hospital.

### Tracheal acquisition

Tracheas were acquired from ten female 6 months adults New Zealand white rabbits (2.5–3.0 kg) using standard surgical procedures. The entire trachea was exposed via a mid-section cervical incision. Next, the sternothyroid muscles were split, the trachea was resected from the larynx to the carina, which was stored in cold PBS with 1% antibiotic and antimycotic solution (AA, Sangon, Shanghai, China).

### Preparation of the decellularized trachea graft

Decellularized rabbit tracheal grafts were obtained by a detergent-enzymatic method. The native trachea was placed in distilled water at 4 °C for 48 h and then incubated with 4% sodium deoxycholate solution (Sigma, USA) for 4 h at 37 °C with a 40 r/min continuous vibration. Subsequently, the native trachea was incubated with 2000 kU/L Dnase-I (Sigma, USA), 1 moL/L NaCl solution at 23 °C for 3 h with a 40 r/min continuous shaking, washed with PBS for 10 min and finally placed in PBS solution containing 1% penicillin, streptomycin and amphotericin B (Sangon, Shanghai, China) overnight. After incubation and rinsing were repeated for seven times, the decellularized trachea was obtained.

### Preparation of the 3D-printed tracheal graft

**Fig. 1:**
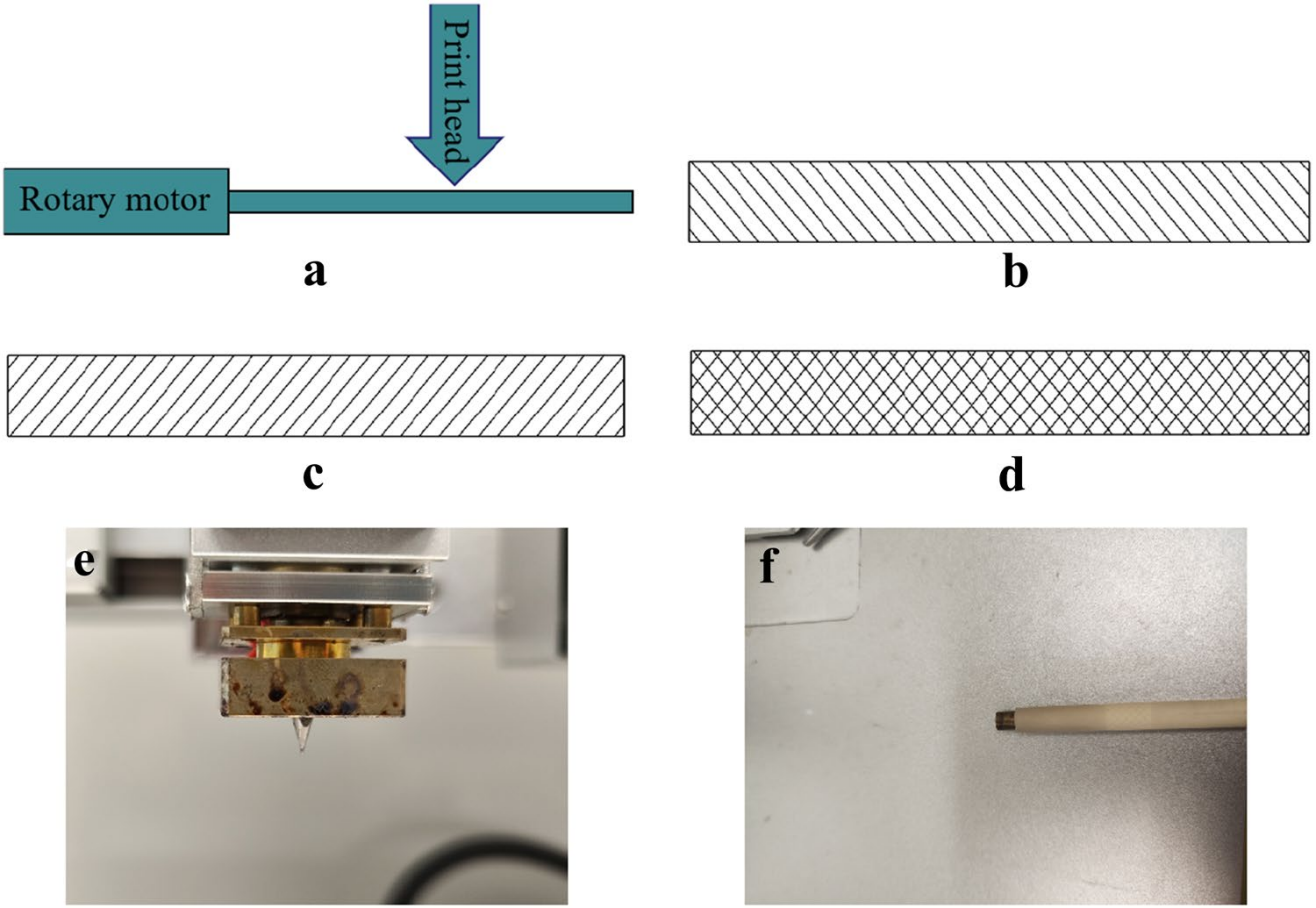
The schematic diagram of 3D-printed tracheal graft (from **a**–**d**). The detailed procedures are described in Preparation of the 3D-printed tracheal graft. **e** Nozzle; f: Rotating shaft

The bio-printer (UN-3DBI-I01) was bought from the Qingdao Unique Products Factory. The 3D-printed tracheal graft was synthesized using PCL (Daixun Trading Co., Ltd., Guangzhou, CAS code: 24980-41-4, molecular weight: 114.142), which was first melted at 90 °C and was extruded out of the nozzle (Fig. 1e, Youni Technology Co., Ltd., Qingdao) to print on the rotating shaft (Fig. 1f) at 5 mm/s (Fig. 1a). The bottom layer of the material was deposited on the platform (Fig. 1b). Once the first layer of material was deposited, another layer was then delivered on top of the previous one from the opposite direction according to the predefined specifications to form a porous shape (Fig. 1c). The process was repeated six times until an ideal tracheal graft was obtained (Fig. 1d). The aperture of the tracheal graft was adjusted by changing the speed of the rotation axis and print head. At last, the graft was immersed in a nano-silica solution (Chemical Engineering College of Yangzhou University) with a concentration of 10% overnight. After five days of condensation, a nano-silica-modified 3D-printed graft was obtained. The 3D-printed tracheal graft thickness was 1 mm, with an inner diameter 6 mm. The interstrand distance was 0.3 mm, and the pore diameter was 200 nm.

### Biomechanical testing of the tracheal graft

The length, diameter, thickness and other morphological data of tracheal grafts in the native tracheal graft group, decellularized tracheal graft group and 3D-printed tracheal graft group were detected based on vernier calipers. A Instron 3367 mechanical testing device (Instron, MU, USA) was applied to examine the sample mechanical properties. The tensile test was performed at a successive rate of 1 mm/s rate with an initial load of 1 N. Similarly, the luminal compression experiment was conducted at a 10 mm/s velocity and an initial load of 0.1 N. Under the condition of the lumen was compressed to half its diameter, the experiment was terminated and the indicators were recorded.

### SEM micrographs of tracheal grafts

The three tracheal grafts (the native tracheal graft group, decellularized tracheal graft group and 3D-printed tracheal graft group) were coated with gold/palladium using a sputter coater for 20 min. Finally, the morphology of the grafts was observed by scanning electron microscope (SEM; Hitachi, Japan). Dehydration was achieved by placing the tissue in graded ethanol (70%, 80%, 90%, 95% and 100%).

### Isolation and culture of mesenchymal stem cells

The BMSCs were isolated from the tibial plateau of 2-months-old female white rabbits (1.5–2.0 kg) and cultured using whole bone marrow adherent culture under aseptic conditions. The rabbit was anesthetized by an intramuscular injection of 0.2 ml/Kg xylazine hydrochloride. Sterile bone marrow puncture needles (Baimusi Precision Technology Co., Ltd. Shenzhen, China) were used to puncture the left and right tibial plateaus, then 2 ml of bone marrow was extracted with a 5 ml syringe containing heparin. The mixture was transferred to a 15 ml centrifuge tube (Corning, NY, USA) on a clean bench. The mixture was centrifuged at 1000 r/min for 5 min (Thermo Fisher, Germany), and the supernatant was discarded. Pellets were washed with PBS. Cells were re-suspended with DEME-F12 medium (Hyclone, USA) with 10% FBS ( Clark, USA). The cell suspension was then transferred to a petri dish and cultured in a 5% CO_2_ incubator (RS232, Thermo Fisher) at 37 °C. The medium was replaced each 48 h, and non-adherent cells were removed. When cells in the culture dish reached 80% confluence, the medium was discarded and washed with PBS twice. Subsequently, cells were re-suspended by 2.5 g/L trypsin (Gibco, NY, USA) at 1 ml. The experiment was repeated to obtain the fourth generation of BMSCs. The fourth-generation BMSCs, which exhibited excellent growth, were used for subsequent experiments.

### In vitro biocompatibility testing

#### Cellular morphology

The experiment includes the following groups: blank control group, native tracheal graft group, decellularized tracheal graft group and 3D-printed tracheal graft group. The tested materials were trimmed to square samples of dimension 1 × 1 cm in a sterile environment and placed into the cell culture plate. 1 ml of the fourth generation of BMSCs cell suspensions (3 × 10^5^ cells/ml) and 1 ml of the BMSCs medium were then added. In addition, in each group, the experiments were repeated three times in parallel. All cell were cultured under the condition of 5% CO_2_ and 37 °C. Finally, cellular morphology on a different part of materials was examined by inverted microscopy (CKX41SF, Olympus).

#### Proliferation index of the cells

CCK-8 method was applied to evaluate the BMSCs proliferation performance. The tested materials were trimmed to obtain square samples of dimension 0.5 × 0.5 cm, immersed in 75% ethanol for 15 min, then treated for 12 h at a distance of 50 cm from the ultraviolet lamp (65w). Ultimately, the materials were put into 10 ml of DMEM-F12 with 10% FBS. The mixture was shaken at 37 °C for 24 h to obtain a steeping medium for the following experiment. The fourth-generation BMSCs were re-suspended in the BMSCs medium and seeded with a density of 3 × 10^4^ cells/ml in a 96-well plate and 100 μL of each well. The culture condition is 5% CO_2_ and 37 °C. After 1 day, the BMSCs medium was replaced by the steeping medium. The CCK-8 assay was analysed on days 1, 3, 5, 7. The absorbance value was detected at a wavelength of 450 nm using a reader (Epoch, BioTek, Vermont, USA). The OD_450_ value which indirectly reflected active cells number was attained after subtracting the absorbance value of the blank control group.

### In vivo experiment: subcutaneous implantation

For in vivo experiments, subjects were classified into three groups: native, decellularized and 3D-printed tracheal graft groups. Fifteen female adults white rabbits (2.5–3.0 kg, 6 months) were obtained from the Medical College of Yangzhou University.

#### Surgical procedure

The experimental animals were anesthetized with Xylazine (0.2 ml/Kg intramuscularly) until the operation was completed. The back was shaved 5 cm around the incision and sterilized with 75% alcohol. The skin and superficial fascia were cut open, and the neck muscles were bluntly separated. Subsequently, the tracheal graft was embedded. Finally, the muscle, fascia were sutured layer by layer after ensuring no leakage or bleeding in the operating field. 50,000 U/Kg of penicillin was injected intramuscularly every day within one week. The rabbits were killed at 30 d after the operation, and the implanted tracheal grafts were obtained.

#### Dynamic analysis of serum immunoglobulin

On days 3, 7, 11, 15, 19, 23 and 27 after the operation, blood samples were collected from the marginal ear veins of the rabbits. ELISA kit (Rapidbio, USA) was used to estimate the dynamic changes in serum IgM and IgG in the rabbits. Hematological parameters (leukocytes, lymphocytes, monocytes, neutrophils) were analyzed by an automatic blood analyzer (Roche, USA).

#### Histological analysis

The samples were fixed with 10% neutral formaldehyde (Zhenxing Chemical Factory, China, pH 7.4) at 23 °C for 24 h and rinsed with distilled water, embedded in paraffin, and cut into 4 mm sections. The tissue sections in the three groups underwent H&E staining (Keygen, Shanghai, China) to assess the growth of tissues, including epithelial tissue, cilia, cartilage and muscle, in terms of kit instructions.

#### Immunohistochemical analysis

The density of macrophages in the tracheal grafts in each group was demonstrated by CD68 immunohistochemical analysis. The regeneration of ciliated and glandular epitheliums was observed. The tissue sections were deparaffinized and hydrated (the steps were the same as HE staining). Subsequently, the samples were incubated with anti-rabbit CD68 (Novus, USA) which was diluted with 1:200 at 4 °C for 12 h. The slides were then incubated with secondary antibodies for 30 min and washed via PBS throughly. Subsequently, staining with DAB was performed for 3 min. Finally, the sample was stained with hematoxylin for 8 min. The stained slides were dried and observed by microscope (Olympus).

Different regions of the tissue sections stained by immunohistochemistry were observed. The cross-section of the trachea was divided into extra-trachea (the area outside the cartilage ring), cartilage matrix and intraluminal regions (the area within the cartilage ring excluding the submucosa). The cell and tissue in the same area of every group were analyzed and compared.

#### Quantification of IL-2 and IFN-γ

Peripheral blood was obtained from the marginal ear vein on days 0 and 7 after BMSCs were co-cultured on tracheal grafts. The IL-2 and IFN-γ concentrations were analysized based on the ELISA method via Rabbit immunoassay kits.

### Statistical analysis

All statistics were analyzed with SPSS 19.0 software,and the data were expressed as mean ± SD. Differences between groups were determined based on ANOVA followed by *t* tests. *p* < 0.05 was regarded as the statistically judgement standard.

## Results

### Morphological observation of tracheal grafts

**Fig. 2:**
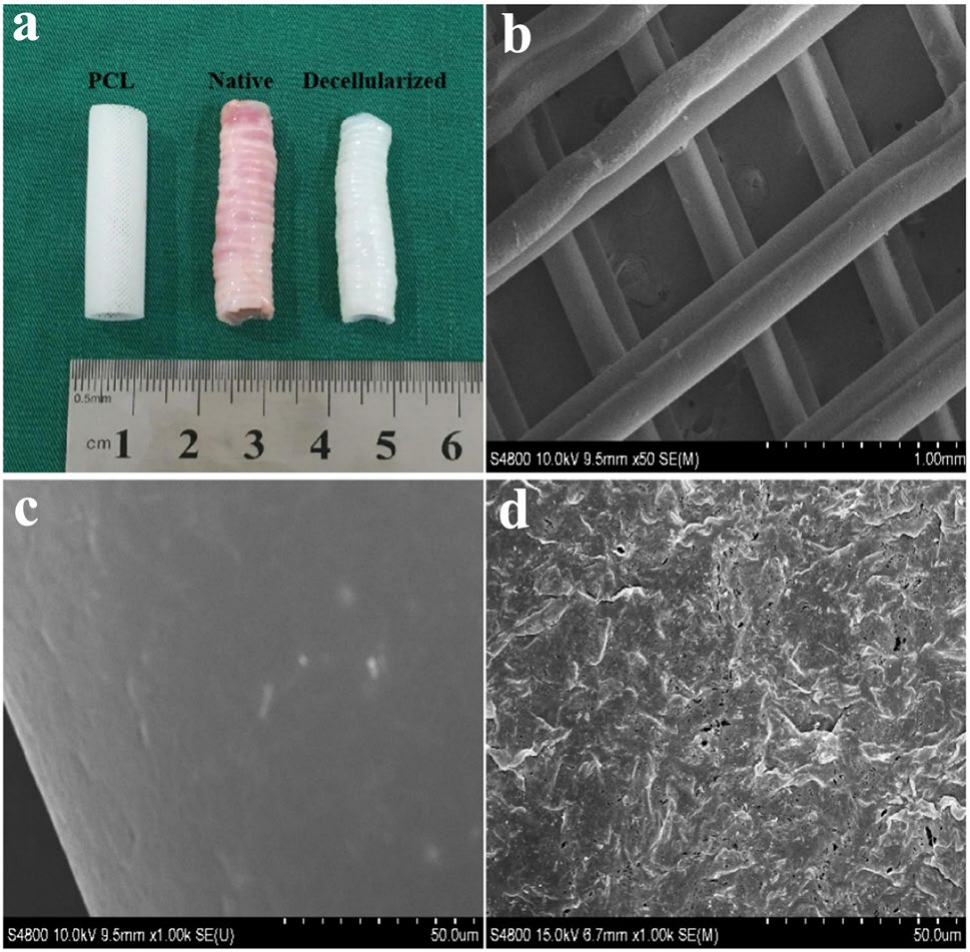
The macroscopic and microscopic structure of tracheal grafts. **a** The macroscopic structure of tracheal grafts; **b** SEM observation of 3D-printed tracheal graft (× 50); **c** SEM observation of 3D-printed tracheal graft (× 1000); **d** SEM observation of decellularized tracheal graft (× 1000). Note: PCL (3D-Printed tracheal graft); Native (Native tracheal graft); Decellularized (Decellularized tracheal graft)

The macroscopic and microscopic structures of the three tracheal graft groups are shown in Fig. 2. The length, lumen inner diameter, and thickness of each group were listed in Table 1. There was no obvious morphological difference among the three tracheal graft groups.

**Table 1:** The macroscopic structures of tracheal grafts (*n* = 5)

Index	Native	Decellularized	PCL
Morphology			
Length (mm)	50.43 ± 0.37	50.30 ± 1.11	50.75 ± 0.38
Width (mm)	8.77 ± 0.35	8.47 ± 0.33	8.80 ± 0.18
Thickness (mm)	0.89 ± 0.04	0.85 ± 0.13	0.88 ± 0.04

### Biomechanical properties of tracheal grafts

The biomechanical properties of the 3D-printed tracheal graft were obviously higher compared to the native and decellularized tracheal grafts. As shown in Table 2, the tensile test results showed that the deformation of the 3D-printed tracheal graft was significantly smaller than that of the other two groups under the same tensile force, revealing that the 3D-printed tracheal graft had the good longitudinal tensile ability. Similarly, The compression test results showed that the maximum stress and elastic modulus of the 3D-printed tracheal graft were better than those of the other two groups when the lumen deformation was 50%, revealing that the 3D-printed tracheal graft had good lateral compression capacity to maintain the lumen shape.

**Table 2:** The biomechanical characteristics of tracheal grafts (*n* = 5)

Index	Native	Decellularized	PCL
Stretched mechanical property			
F max [*N*]	8.03 ± 0.50	7.71 ± 0.36	28.96 ± 0.54^a,b^
Tensile strain at break (mm/mm)	0.21 ± 0.03	0.20 ± 0.01	0.18 ± 0.03
Elastic modulus (mPa)	4.58 ± 0.09	4.53 ± 0.26	45.55 ± 6.64^a,b^
Tensile strength (mPa)	0.69 ± 0.20	0.67 ± 0.24	4.44 ± 0.29^a,b^
Compressive mechanical property			
Loading (*N*) of 50% deformation in compression	2.29 ± 0.14	1.30 ± 0.14^a^	5.00 ± 0.01^a,b^
Elastic modulus (N/mm^2^)	0.56 ± 0.16	0.28 ± 0.06^a^	1.72 ± 0.23^a,b^

ANOVA, compared with the native tracheal graft (^a^*p* < 0.05), decellularized tracheal graft (^b^*p* < 0.05)

### The biocompatibility testing of cell-tracheal grafts in vitro

#### Culture and passage of BMSCs

**Fig. 3:**
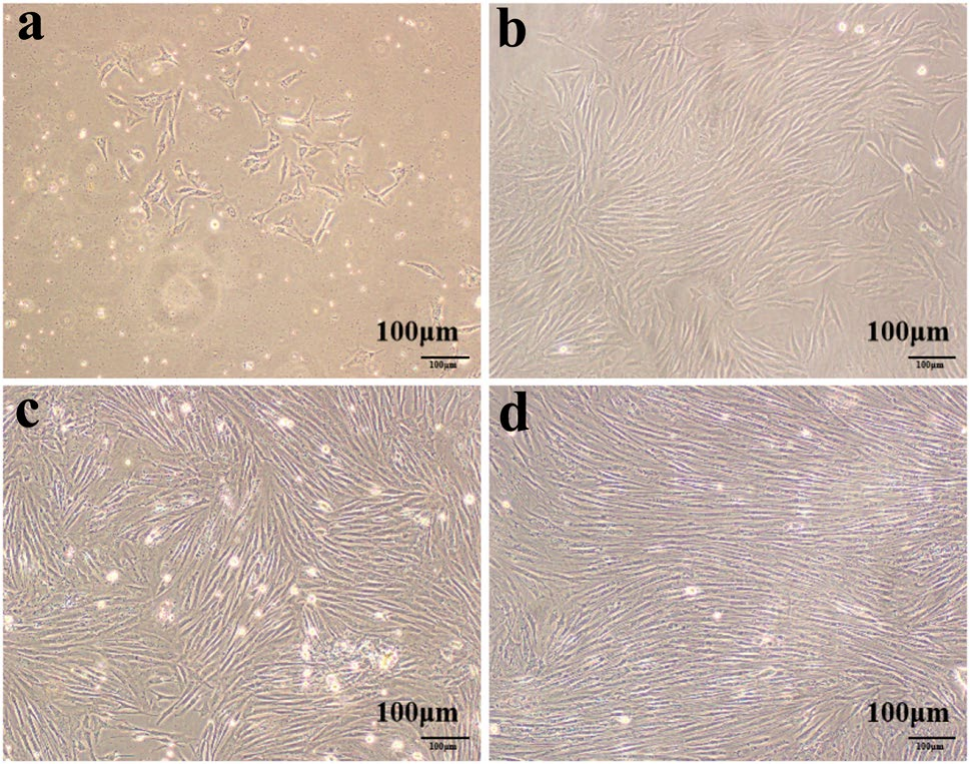
Morphological observation of BMSCs. Scale bar:100 μm. Note: **a** After 48 h culture; **b** After 6d culture; **c** After 10d culture; **d** The 4rd passage BMSCs

The results showed that BMSCs grew in clusters with different shapes, mainly fusiform after 48 h (Fig. 3a). At 6 d, the BMSCs colony was formed and the cell morphology was mostly fusiform and polygonal (Fig. 3b). At 10 d, BMSCs reached 90% fusion (Fig. 3c). The proportion of flat cells in the 4th generation BMSCs were increased (Fig. 3d).

#### Cell morphology observation

**Fig. 4:**
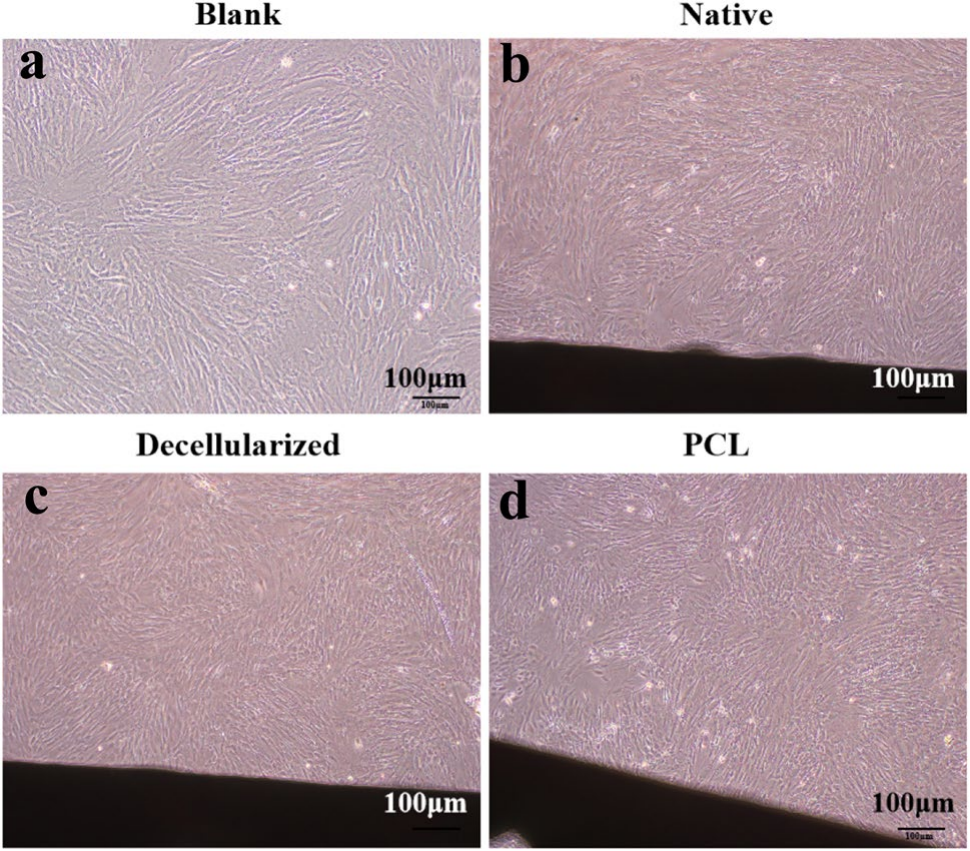
Observation of three tracheal graft groups co-cultured with BMSCs for 7d. Scale bar:100 μm. Note: **a** Blank control group; **b** Native tracheal graft group; **c** Decellularized tracheal graft group; d: 3D-printed tracheal graft group

After co-culturing for 7 d, the results showed that BMSCs in the three groups grew well and adhered to the tracheal grafts. There was no significant distinction in the morphology of BMSCs around three groups of tracheal grafts, and few suspended BMSCs were appeared in the 3D-printed tracheal graft group (Fig. 4). There was no significant distinction in the cell density of the three groups of tracheal grafts.

#### Results of the CCK-8 assay

**Fig. 5:**
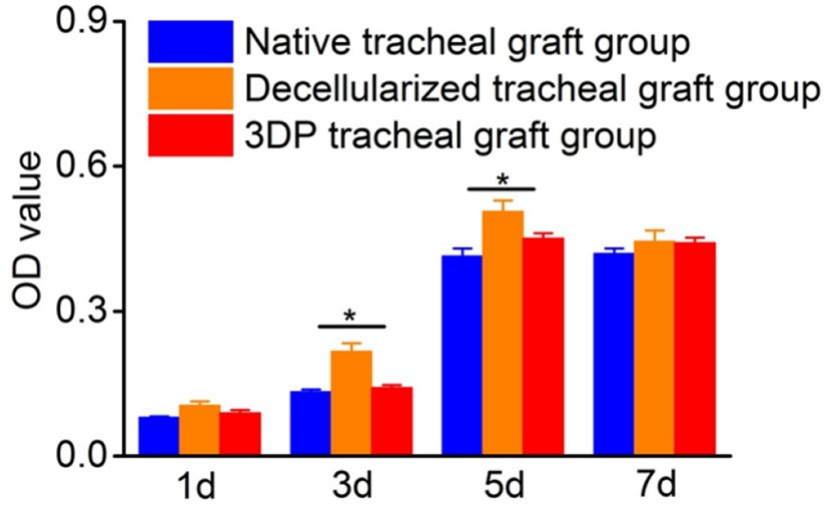
Cell proliferation in three groups of tracheal grafts by CCK-8 (*n* = 3). Note: Three tracheal graft groups grew with BMSCs

The proliferation of cells co-cultured with tracheal grafts in all three groups was measured with a microplate reader (450 nm wavelength) on days 1, 3, 5, and 7. As shown in Fig. 5, In the beginning week, the cells attached to the material surface and proliferated rapidly, and the number increased approximately 5 times on the 5th day. For reasons of no subsequent passage, self aging and death, the cell number decreased at 7 days. However, the OD values for cells in the decellularized tracheal graft group were statistically higher compared to the other two groups on days 3 and 5 (*p* < 0.05).

#### Postoperative observation

**Fig. 6:**
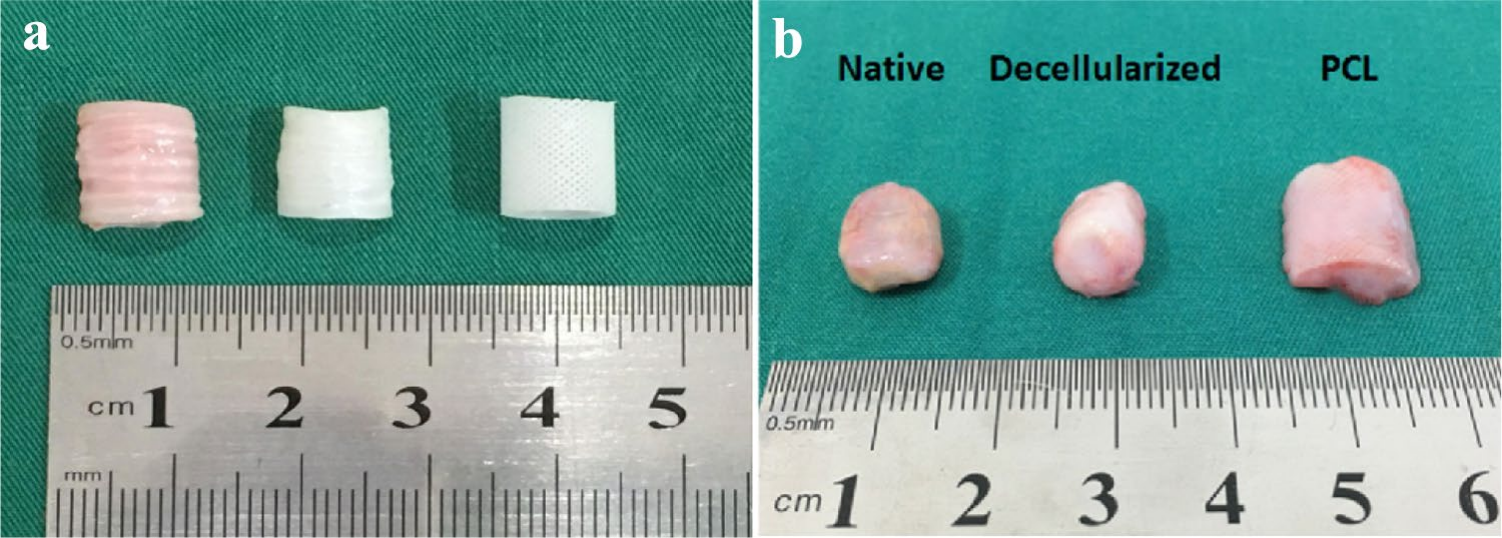
Comparison of three groups of tracheal grafts before and after implantation. (**a** Before implantation; **b** After implantation)

All the recipient animals showed good general health conditions, weight gain and wound healing within 30 days after the operation (Fig. 6). After 30 days, the lumen of the native tracheal group collapsed. The structure was destroyed and wrapped to form cystic inclusions, with abundant pus accumulated in the cysts. In the decellularized tracheal group, the lumen collapsed and adhered to the recipient bed, which was difficult to peel off. That of the 3D-printed tracheal group was well maintained, wrapped by inflammatory tissue and easy to peel off.

#### Detection of immunoglobulin

**Fig. 7:**
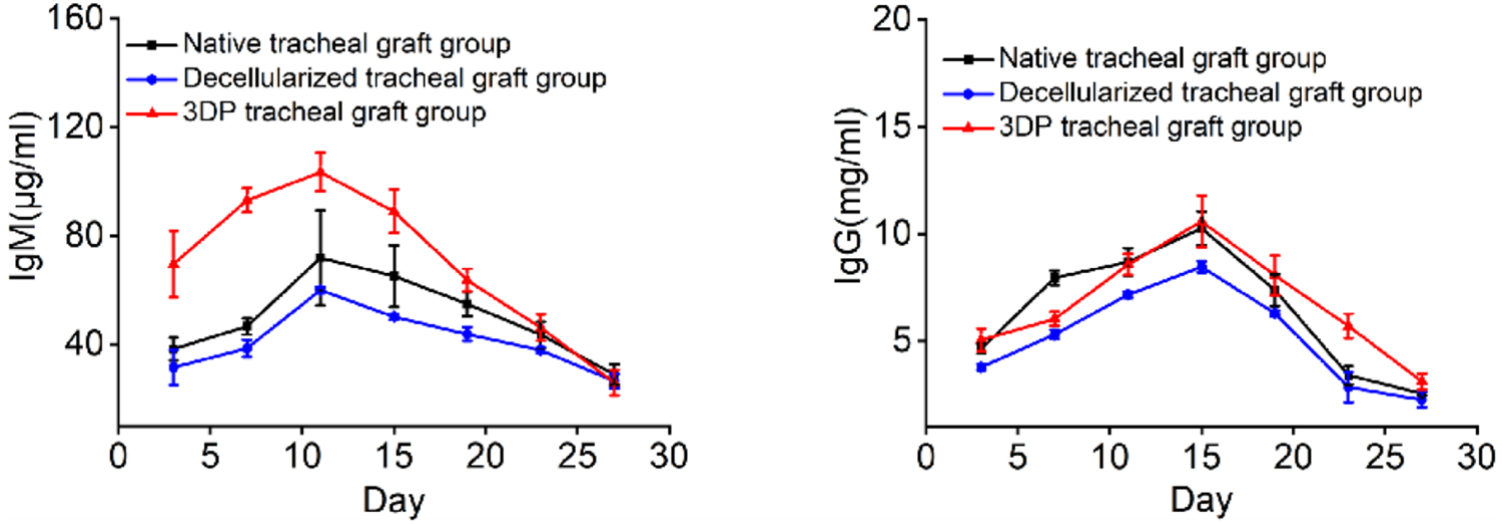
In vivo evaluation of IgG and IgM levels of allogeneic transplant recipients (*n* = 5)

As shown in Table 3 and Fig. 7, the IgM and IgG level of the three groups increased gradually, reached a peak on days 11 and 15, respectively, and then decreased gradually. The contents of IgM and IgG in the 3D-printed tracheal group were obviously higher than in the decellularized tracheal group after the operation (*p* < 0.05).

**Table 3:** In vivo evaluation of IgG and IgM levels of allogeneic transplant recipients (*n* = 5)

Days	Native		Decellularized		PCL	
	IgM (μg/ml)	IgG (mg/ml)	IgM( μg/ml)	IgG (mg/ml)	IgM (μg/ml)	IgG (mg/ml)
3	38.30 ± 4.13	4.65 ± 0.22	31.57 ± 6.45	3.79 ± 0.14^a^	69.63 ± 12.24^a,b^	5.06 ± 0.53^b^
7	46.66 ± 2.99	7.95 ± 0.34	38.58 ± 3.18^a^	5.31 ± 0.21^a^	93.12 ± 4.46^a,b^	6.04 ± 0.33^a,b^
11	71.86 ± 17.51	8.69 ± 0.64	60.05 ± 1.14	7.17 ± 0.14^a^	103.48 ± 7.08^a,b^	8.57 ± 0.50^b^
15	65.24 ± 11.37	10.27 ± 0.79	50.20 ± 1.13^a^	8.46 ± 0.27^a^	89.08 ± 8.00^a,b^	10.59 ± 1.20^b^
19	54.97 ± 4.54	7.38 ± 0.75	43.78 ± 2.59^a^	6.30 ± 0.14^a^	63.71 ± 4.09^a,b^	8.07 ± 0.92^b^
23	43.59 ± 4.87	3.41 ± 0.43	37.90 ± 1.09^a^	2.85 ± 0.71	46.19 ± 4.90^b^	5.70 ± 0.57^a,b^
27	28.97 ± 3.73	2.55 ± 0.08	26.53 ± 2.56	2.27 ± 0.36	25.95 ± 4.73	3.12 ± 0.38^a,b^

ANOVA, compared with the native tracheal group (^a^*p* < 0.05), compared with the decellularized tracheal group (^b^*p* < 0.05) at the same time point

#### Detection of blood routine

As shown in Table 4, the white blood cells reached a peak about a week after the operation and then began to decline. The neutrophils number gradually reduced, while lymphocytes increased, and monocytes kept stable. In the native tracheal graft group, the white blood cells components were dominantly neutrophils in the 2 weeks after surgery, while in the 3D-printed tracheal group, the number of neutrophils was less than lymphocytes in 1 week after surgery. In the decellularized tracheal group, the number of white cell was fluctuated, while the total white cell level was obviously lower than that of the other two groups (*p* < 0.05).

**Table 4:** Results of blood routine examination (*n* = 5)

Days	Native				DEM				PCL			
	Leukocyte (× 109/L)	Neutrophils (%)	Lymphocytes (%)	Monocytes (%)	Leukocyte (× 109/L)	Neutrophils (%)	Lymphocytes (%)	Monocytes (%)	Leukocyte (× 109/L)	Neutrophils (%)	Lymphocytes (%)	Monocytes (%)
3	9.59 ± 0.90	58.91 ± 9.08	30.83 ± 11.84	3.45 ± 0.39	7.77 ± 0.38^a^	55.27 ± 6.55	39.55 ± 6.72	4.32 ± 0.20^a^	10.03 ± 0.61^b^	57.28 ± 6.01	39.21 ± 3.40	3.41 ± 0.20^b^
7	11.79 ± 1.36	57.22 ± 4.11	39.42 ± 4.25	3.15 ± 0.45	9.47 ± 0.18^a^	71.37 ± 2.63^a^	26.60 ± 1.08^a^	3.68 ± 0.48	10.28 ± 1.26	42.06 ± 2.96^a,b^	54.94 ± 2.70^a,b^	3.26 ± 0.07
11	9.71 ± 0.48	55.07 ± 2.31	44.00 ± 4.09	3.67 ± 0.75	8.02 ± 0.15^a^	60.18 ± 2.22^a^	35.67 ± 2.23^a^	3.82 ± 0.09	10.11 ± 0.98^b^	40.66 ± 2.52^a,b^	54.52 ± 1.43^a,b^	3.69 ± 0.85

ANOVA, compared with the native group (^a^*p* < 0.05), compared with the decellularized group (^b^*p* < 0.05) at the same time point

#### Analysis of HE

**Fig. 8:**
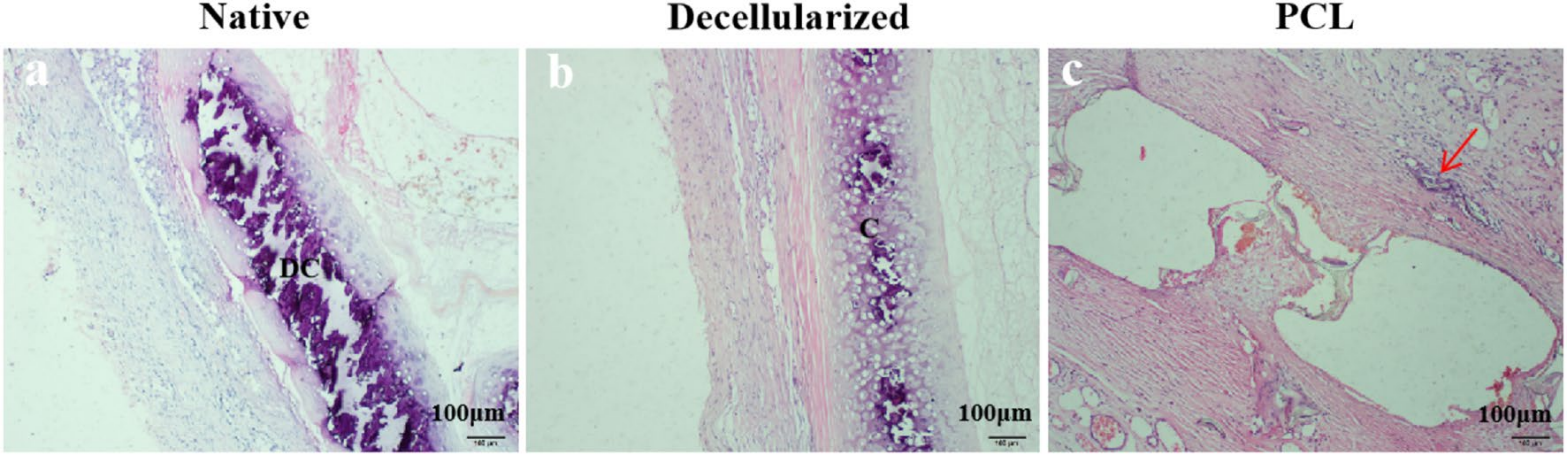
Hematoxylin/eosin (HE)-stained three groups of tracheal grafts on day 30 after implantation. Scale bar:100 μm. Note: **a** Native tracheal graft group; **b** Decellularized tracheal graft group; **c** 3D-printed tracheal graft group; DC(damaged cartilage); C(cartilage); Red arrow (neovascularization around the material)

There were significantly more inflammatory cell infiltrates in the native tracheal group, consisting mainly of monocytes and lymphocytes; the gland structures were not visible, while the cartilage tissue was destroyed to different degrees. For the 3D-printed tracheal group, eosinophil infiltration was the predominant manifestation, showing foreign body granuloma-associated inflammatory reaction and normal gland architecture. The least number of inflammatory cells were infiltrated in the decellularized tracheal group, chondrocytes were of normal morphology, and no calcification and rejection were found (Fig. 8).

#### Analysis of immunohistochemistry

**Fig. 9:**
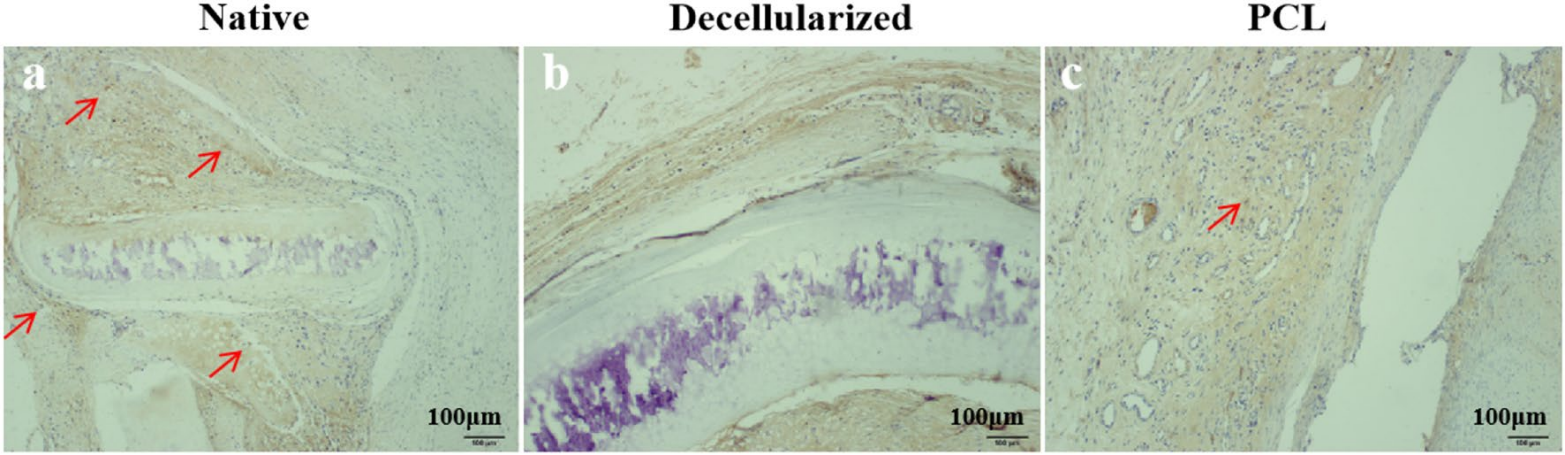
Immunohistochemical analysis of three groups of tracheal grafts on day 30 after implantation showing the presence of CD68 antigen. Scale bar:100 μm; Red arrow represents macrophage. Note: **a** Native tracheal graft group; **b** Decellularized tracheal graft group; **c** 3D-printed tracheal graft group

30 d after the operation, the matrix of the native tracheal group was destroyed. The nucleus in the cartilage depression disappeared, the cell membrane was brown, and abundant CD68-positive macrophages were visible at the submucosa. The structure of the 3D-Printed trachea group remained intact, and CD68-positive macrophage infiltration was seen inside the lumen. Macrophage infiltration in the decellularized tracheal group was significantly less than in the other two groups (Fig. 9).

#### Serum IL-2 and IFN-γlevels

**Fig. 10:**
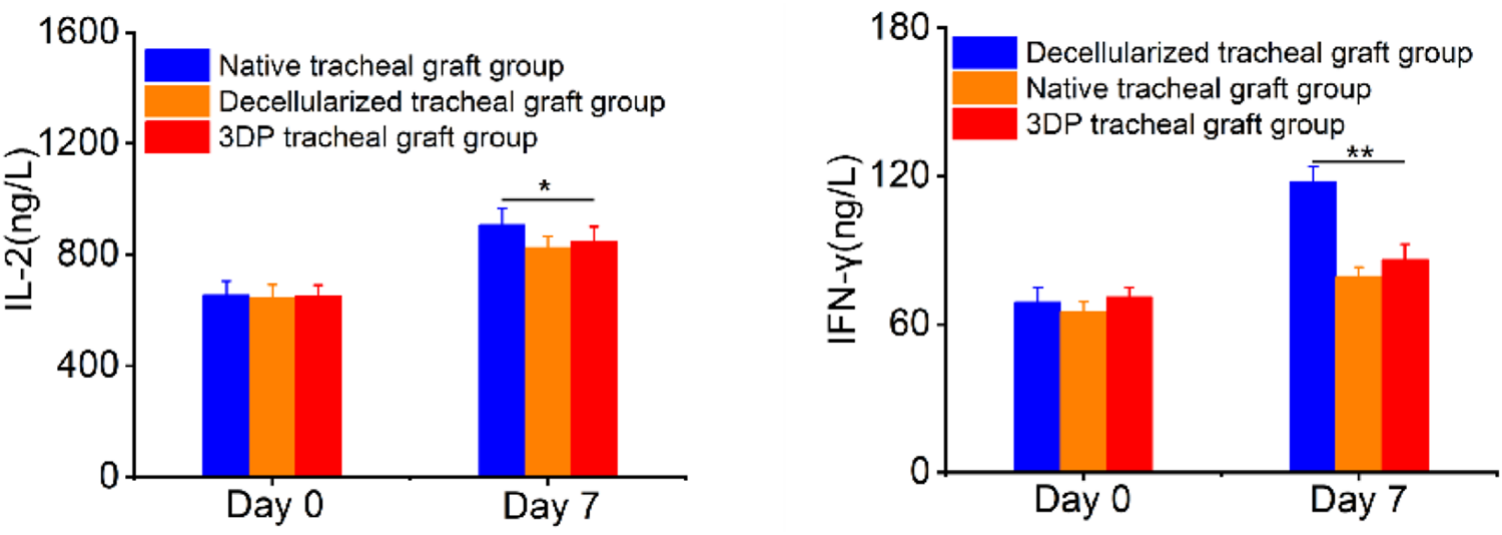
The levels of IL-2 and IFN-γ in peripheral blood in three groups of tracheal grafts on day 7 after implantation (*n* = 5)

On day seven after transplantation, the levels of IL-2 in the native, decellularized and 3D-printed tracheal graft groups were 906.08 ± 26.64, 832.23 ± 13.96, 844.45 ± 16.53 ng/L, respectively. The levels of IFN-γ in the native, decellularized and 3D-printed tracheal graft groups were 118.29 ± 2.45, 80.58 ± 2.71, 83.55 ± 5.47 ng/L, respectively. The expression of IL-2 and IFN-γ in the native tracheal graft group was obviously higher (Fig. 10).

## Discussion and conclusion

Extracellular matrix (ECM) is an important part of tissue engineering for it is very necessary for the survival of seed cells. It strongly influences seed cell adhesion, differentiation, and promotes tissue regeneration and reconstruction [18, 19]. At present, both acellular matrix-based and biosynthetic scaffold-based approaches have been widely used in tissue engineering tracheal research. Acellular matrix materials contain extracellular matrix components and do not elicit inflammatory responses [20, 21]. But the decellularization in vitro is limited to a certain extent by the long duration of the decellularization treatment cycle, many processing links, consumption of manpower, material resources, amd greater risk of contamination [22]. The 3D-Printed tracheal stent brings many advantages, including individualization, precision, synchronization between seed cells and scaffold materials and rapidity [23].

It has been established that the reconstructed airways should possess the appropriate form and robustness to maintain their shape [24]. If the shape cannot be maintained, collapse and stenosis may occur during the early postoperative period. In the present study, through the mechanical performance test, it was found that the 3D-Printed tracheal graft had significant advantages compared with the decellularized tracheal graft. However, the poor acellular biomechanical properties were thought to be related to the tracheal loss of soluble collagen.

It should be borne in mind that for a tissue-engineered tracheal graft material to be applied in clinical practice, it should support seed cell adhesion, proliferation, and differentiation. In this experiment, the outer wall of two tracheal grafts was seeded with BMSCs for co-culture in vitro, and the adhesion of cells were observed. Accordingly, our findings suggest that decellularized and modified 3D-Printed tracheal grafts can offer a good cell adhesion interface for cell proliferation. The relatively greater number of cells attached to the 3D-Printed tracheal graft surface was attributed to the suitable pore size of the material and increased number of attachment points available for adhesion on original hydrophobic material surface after modification. Results from co-culture in vitro and the CCK-8 assay suggested that the least cytotoxicity was observed in the decellularized tracheal graft group, which provided the most conducive environment for cell proliferation. Notwithstanding that 3D-Printed tracheal graft facilitates cell adhesion, cell growth, it is associated with a lower proliferation rate of BMSCs compared to the decellularized tracheal graft.

Biomaterials should not satisfy the functional requirements but also be biocompatible for long-term or short-term contact with organisms. In this study, serum IgG and IgM changes were monitored after the operation, and HE staining and immunohistochemical staining for macrophage-specific antigen CD68 were performed after obtaining the specimens. In addition, The IL-2 and IFN-γ concentrations were measured 7d after the operation. Our results showed that IgM peaked first, while IgG peaked after two weeks, consistent with the characteristics of these two immunoglobulins. IgG and IgM levels in the 3D-Printed tracheal group were obviously higher compared to that in other groups at 2 weeks. The results of the three groups were consistent at four weeks, indicating that the 3D-Printed tracheal group induced higher levels of stimulation than in the other two groups during the acute stage of inflammatory reaction. IL-2 and IFN-γ are mainly released by activated T lymphocytes, which are effective markers of rejection induced after transplantation [25]. The experimental results show that IL-2 and IFN-γ levels were increased in three groups. However, the expression of the two in the native tracheal graft group was higher compared to the two other groups. The production of IL-2 and IFN-γ is increased which indicates acute rejection during transplantation [26]. HE staining showed significant infiltration of mononuclear macrophages, lymphocytes and plasma cells in the native tracheal group, while a predominant infiltration of eosinophils was appeared in the 3D-Printed tracheal group, showing foreign body granulomatous inflammation. Given that the inflammatory reactions induced are completely different, the relationship between the antigen and the material’s surface should be considered. Since there is no antigen on the surface of the 3D-Printed tracheal scaffold, the antigen presentation process cannot occur, leading to fibrous encapsulation and foreign body granuloma. In addition, eosinophil infiltration may occur, resulting from autoimmune reactions caused by foreign body stimulation. To sum up, by analyzing the biological performance of decellularized tracheal scaffold and 3D-printed tracheal scaffold in vitro and in vivo in this experiment, we confirmed that the 3D-printed tracheal scaffold exhibited relatively better biomechanical properties. However, the decellularized trachea exhibited low immunogenicity, adequate cell proliferation and good biocompatibility. Indeed, more studies are required to improve our knowledge on the advantages of these two scaffold materials to provide the basis for constructing hybrid tissue-engineered tracheas with multi-layer structures.

## Data Availability

The original contributions presented in the study are included in the article. Further inquiries can be directed to the corresponding authors.
